# Psychometrics properties of the Iranian version of fertility quality of life tool: A cross- sectional study

**Published:** 2018-03

**Authors:** Seyedeh-Fatemeh Hekmatzadeh, Fatemeh Bazarganipour, Nazafarin Hosseini, Helen Allan, Somayeh Jalali, Zahra Abbasian, Akram Barani, Fereshteh Balochi, Saeideh Khademi, Tahereh Mahmoudi, Roghayeh Niknam, Zahra Khashavi, Seyed Abdolvahab Taghavi

**Affiliations:** 1 *Social Determinants of Health Research Center, Yasuj University of Medical Sciences, Yasuj, Iran.*; 2 *Centre for Critical Research in Nursing and Midwifery, School of Health and Education, Middlesex University, London, UK.*; 3 *Student Research Committee, Hormozgan University of Medical Sciences, Bandar Abbas, Iran.*; 4 *Hazrate-zahra Infertility Center, Bandar Abbas, Iran.*

**Keywords:** Fertility, Quality of life, Validity, Reliability, Iran

## Abstract

**Background:**

Clinical measurement of quality of life (QoL) for assessing reproductive problems should be considered as a standard investigation at the initial and continuing medical consultations with infertile people.

**Objective:**

The purpose of this study was comprehensive testing the psychometric properties of the Iranian version of fertility quality of life (FertiQoL).

**Materials and Methods:**

This cross-sectional study was conducted on300 women referred to infertility clinic. After linguistic validation, a semi-structured interview was conducted to assess face validity. Consequently exploratory factor analysis was performed to indicate the scale constructs. Discriminate validity was assessed using the known groups comparison. Convergent validity was evaluated by assessing the correlation between similar content on the 12-Item Short Form Health Survey (SF12), Hospital Anxiety and Depression Scale and FertiQol. In addition, reliability analysis was carried out with internal consistency.

**Results:**

The reliability of the Iranian version of the FertiQoL was satisfactory in all dimensions (0.77-0.83). Six factors (emotional, mind/body, relational, social, environmental, and tolerability) were extracted from the results of exploratory factor analysis. Discrimination validity showed that FertiQoL can differentiate between female patients with differing duration of infertility and number of children. Moreover, the results of convergent validity showed a favorable correlation between the related dimensions of SF12 (0.43-0.68), Hospital Anxiety and Depression Scale (0.47-0.52) and FertiQoL.

**Conclusion:**

The Iranian version of FertiQoL is valid and reliable for assessing infertility problems and the effects of treatment on QoL of infertile patients referred for diagnosis and treatment at infertility clinic.

## Introduction

In Iran, as in many developing countries, fertility as a social value and a necessary condition for married women considered. So, infertility usually as unpleasant features intended for couples. The sensitivity of childlessness is also evident in the support of Iranian families. According to Article nine of the law, infertility can justify the dissolution of marriage by divorce (1). In Iranian culture, patriarchal beliefs to the need to reproduce, lack of social and financial support for many women, the chances of a second marriage for infertile women and social opposition of single-lives are some of the factors that may be caused to the mental suffering of infertile women (2).

Health-related quality of life (QoL) is currently considered as a major tool for measuring outcome in infertile couples. Given the different physical, psychological, and social adverse effects of infertility, evaluating the components of QoL in these couples may lead to identify different aspects of lifestyle in these populations and help them to planning appropriate treatment with greater efficiency (3, 4). In addition, despite development of various techniques for treatment of infertility and achieving reproductive health, concerns about QoL in infertile couples has been clearly decreased due to the nature of the problem as well as to its complex relationship with mental status (5, 6).

Clinical measurement of quality of life for assessing reproductive problems should therefore be considered as a standard investigation at the initial and continuing medical consultations with infertile people. Boivin and colleagues have developed a specialized tool for the measurement of fertility quality of life (FertiQoL) (7). This questionnaire has been translated into 20 languages around the world and is considered valid and reliable in those versions. Although the Iranian version of the questionnaire is available, Maroufizadeh and colleagues have found the psychometric properties of the Iranian version of the questionnaire (8). Although there are some notices that needed to more attention. This study sample size is 120 participates considering 5 patients were necessary for each item (subject-to-item ratio: 5:1 ((8). However, the FertiQoL have 36 questions and at least 180 participants are needed for evaluation of psychometrics properties.

Therefore, the purpose of this study was comprehensive testing the psychometric properties of the Iranian version of FertiQoL as its use could be considerable due to the prevalence of infertility in Iran and the importance of evaluation of QOL in patients with infertility.

## Materials and methods

**Design and data collection**

This cross-sectional study was conducted on 300 women who referred to the Omeleila infertility clinic (only referral infertility clinic in Hormozgan), Hormozgan, Iran between April 2015 to September 2016. The inclusion criteria for the women were being married, have a desire to participate in the study, age range between 15-45 yr, have been diagnosed with infertility by an experienced physician, having no severe psychological experiences over the past six months (loss of a close relative, surgery), and using no psychiatric medications at the time of participation in the study. After explaining the purpose of the study and obtaining written consent from participants who were eligible to enter the study, women were asked to complete the questionnaire in the waiting room before their medical consultation. The average time taken to complete the questionnaires was 30 min.

FertiQoL questionnaire is a self reported measure for assessment of QoL? in infertile patients developed by the European Society of Human Reproduction and Embryology and the American Society for Reproductive Medicine. The questionnaire consists of two general questions 1) how would you rate your health? 2) Are you satisfied with your quality of life? And two modules measuring QoL 3) optional treatment 4) core module. The core module included 24 specific questions about fertility and consisted of four domains (emotional, social, mind/ body, relation) with six questions in each field, the optional treatment module measures quality of life in relation to infertility treatment and consisted of two domains, environmental and toleration for infertility treatment. The answers are rated 0-4 based on a Likert scale. The score of each field and the total score were collated and converted to a 0-100 spectrum based on the questionnaire grading instructions. The higher scores indicated better quality of life (7).

**Convergent validity**

It is assumed that similar domains of different tools show a high level of correlation (9). For testing convergent validity, it is assumed that there is a significant correlation between the similar domains of 12-Item Short Form Health Survey (SF12), Hospital Anxiety and Depression Scale (HADS) and FertiQoL. Correlation >0.4 was considered desirable with the following measures (10). HADS questionnaire: this questionnaire includes 14 questions consisted of two subscales of depression (seven questions) and anxiety (seven questions). Grading is based on a Likert scale (0-3) for each component. Therefore, the score of the two subscales is in the range of 0-21 (11). The validity and reliability of this questionnaire are approved in Iran (12).

SF12: This questionnaire was used to evaluate quality of life. SF12 includes 12 questions related to the eight domains of vitality, physical functioning, bodily pain, general health perceptions, physical role functioning, emotional role functioning, social role functioning, and mental health. The scoring is based on the RAND system from zero to 100. The score of each domain is obtained by aggregating the scores of every domain and dividing the resulting number by the number of questions in the same domain. A higher score indicates better quality of life (13). The validity and reliability of the questionnaire are approved for the Iranian setting (14). 

**Discrimination validity**

Measurement of this validity indicates to what extent a tool can differentiate between different groups that are different in special features. In this study, discrimination validity assessment was evaluated by considering to what extent FertiQoL could differentiate between groups of patients who are different in terms of infertility duration and the number of children.

**Factorial validity**

In order to discover underlying variables of FertiQoL, we used exploratory factor analysis. First, the Kaiser-Mayer-Olkin test of adequacy index was completed where the value of greater than 0.7 was considered acceptable. Correlation between the questions was determined using the Kruit-Bartlett test. We used specific value to determine the number of constructor factors of FertiQoL. Varimax rotation was used to simplify data and the interpretation of factor component. After rotation, factors were nominated according to the questions existing in each factor. The factors which loaded greater than 0.3 were used for factor preservation (15). 

In order to reduce statistical error, it is suggested that the number of subjects per question has at least a ration of 5:1. FertiQoL have 36 questions and at least 180 participate are needed for evaluation of psychometrics properties. Therefore, for reduced statistical error, the number of 300 patients was considered suitable to achieve this ration in this study.

**Reliability**

Reliability predicts the consistency and stability of a measure. In the present study, reliability was determined using an internal consistency method. In order to determine internal consistency, Cronbach’s alpha was calculated for the whole questionnaire and also for each of the factors. Cronbach’s alpha >0.7 was considered desirable (16).

**Ethical consideration**

The Ethics Committee of the Yasuj University of Medical Sciences reviewed the study (IR.YUMS.REC.1392.30). A written informed consent was obtained from each participants who was asked to complete the measures. All participants’ information remained confidential.

**Statistical analysis**

Data analysis was completed using SPSS 21 (Statistical Package for the Social Sciences, version 21.0, SPSS Inc, Chicago, Illinois, USA) for both descriptive and inferential statistics methods. For descriptive statistics, the mean±SD and number (%) were used for quantitative and qualitative variables respectively. Intergroup comparison for quantitative variables was done using *t*-test. The significant level of p<0.05 was considered acceptable.

## Results

**The study sample**

300 women with infertility entered the study during a six months period. The mean age of them was 30.65±5.43 yr. Clinical and demographic characteristics of participants are presented in Table I.

**Score of FertiQoL**

The score distribution of FertiQoL and SF12 are presented in Table II. The lowest scores were allocated to emotional domain of FertiQoL and mental subscale component of SF12. Comparing floor and ceiling effect between the two questionnaires shows that the percentage of respondents with minimum and maximum scores to FertiQoL was less than SF12 (Table II).

**Convergent validity**

The correlation between the emotional domain of FertiQoL and depression (p<0.001, r=0.47) and anxiety (p=0.001, r=0.52) domain of HADS was desirable. Moreover, the emotional domain of FertiQoL showed a desirable correlation with the role limitations due to emotional problems (p<0.001, r=0.44), mental health (p<0.001, r=0.43) and vitality (p<0.001, r=0.50) in SF12 questionnaire. 

The social domain of FertiQoL showed a favorable correlation with the social functioning domain of SF12 (p<0.001, r=0.49). In addition, the correlation between the mind/body domain of FertiQoL and role limitations due to physical problems (RP) (p<0.001, r=0.47), physical functioning (p<0.001, r=0.68) was desirable. 

**Discrimination validity**

FertiQoL can differentiate well between infertile women who are different in respect of infertility duration and number of children. The results of table III show that women with more children and shorter duration of infertility have a better quality of life.

**Factor analysis**

The Iranian version of FertiQol was analyzed using principal component factor analysis with varimax rotation. The value of Kaiser-Mayer-Olkin test, an indicative of sample quality, was 0.89. Factor analysis of FertiQol revealed six factors that are responsible for 52.91% of variance: emotional (17.24%), mind/body (10.76%), relational (10.52%), social (5.52%), environmental (4.59%), tolerability (4.26%). Table IV shows the factors loaded in each question. Factor loading was more than 0.3 in all questions, so all questions were preserved.

**Reliability**

Cronbach’s alpha was 0.82 for the whole questionnaire indicating a desirable reliability for FertiQol. Data related to reliability of all fields of FertiQol is presented in Table II. 

**Table I:** Demographic and clinical characterizes of participants

**Variables **		**Mean ± SD**
Age (yr)*		30.65 ± 5.93
Education (yr)*		11.41 ± 4.15
Marriage (yr)*		7.64 ± 5.03
BMI (kg/m^2^)*		25.63 ± 11.83
Cause of infertility		n (%)
	Tubal factors	21 (7)
	Uterus	10 (3.3)
	PCOS	63 (21.1)
	POF	1 (0.3)
	Ovulation dysfunction	31 (10.4)
	Endometriosis	3 (22)
	Unexplained	132 (44.1)
	Male infertility	132 (44.1)

BMI: Body mass index

PCOS: Polycystic ovary syndrome

POF: Premature ovarian failure

**Table II:** FertiQoL and SF12 scores, Cronbach’s alpha, and floor and ceiling effects

	**Subscales**	**Cronbach’s alpha**	**Mean ± SD** **of FertiQoL and SF12 scores**	**Minimum (% floor)**	**Maximum (% ceiling)**
FertiQoL					
	Emotional	0.77	53.53 ± 19.05	4.6 ( 0.3)	99.84 (0.3)
	Mind/body	0.78	62.07 ± 20.83	0 (0.3)	78.08 (0.3)
	Relational	0.79	61.16 ± 17.06	12.48 (0.3)	99.84 (22)
	Social	0.77	57.61 ± 17.68	20.80 (0.3)	99.84 (22)
	Environmental	0.83	57.69 ± 15.74	4.16 (0.7)	95.68 (0.7)
	Tolerability	0.79	59.33 ± 20.18	0 (0.7)	100 (0.3)
	Total	0.82	59.73 ± 13.48	22.88 (0.3)	92.93 (1.3)
SF12					
	Physical functioning	-	67.51 ± 31.52	0 (5.7)	100 (12.3)
	Role limitations due to physical problems	-	56.34 ± 25.32	0 (1.7)	100 (38.7)
	Role limitations due to emotional problems	-	54.41 ± 25.25	0 (1.7)	100 (3.7)
	Vitality	-	54.58 ± 25.98	0 (1.7)	100 (12.3)
	Social functioning	-	39.33 ± 32.02	0 (3.7)	100 (6)
	Bodily pain	-	65.62 ± 29.84	0 (19.3)	100 (26)
	General health perception	-	46.92 ± 30.91	0 (1.7)	100 (8.7)
	Mental health	-	58.06 ± 16.77	0 (0.3)	100 (3)
	Physical subscale component	-	59.19 ± 19.57	5.63 ( 0.3)	100 (2)
	Mental subscale component	-	51.59 ± 11.07	15.63 (3)	87.50 (3)

FertiQoL: Fertility quality of life

SF12: 12-Item Short Form Health Survey

**Table III:** Discrimination validity: Scores in subscales of FertiQoL in different groups of infertile women

**Groups **		**Domains of FertiQoL**					
		**Emotional**	**Mind/body**	**Relational**	**Social**	**Environmental**	**Tolerability**
Child							
	None	52.02 ± 18.67	60.38 ± 19.51	60.62 ± 17.01	63.14 ± 17.37	57.64 ± 16.18	58.13 ± 20.17
	≥1	59.60 ± 19.47	68.99 ± 24.53	63.45 ± 16.89	70.50 ± 18.85	59.24 ± 13.88	64.25 ± 19.64
	p-value	0.001	0.005	0.2	0.004	0.49	0.04
Duration of infertility							
	<1 yr	53.82 ± 19.38	63.28 ± 20.47	61.57 ± 17.11	65.18 ± 17.77	57.37 ± 15.75	59.28 ± 20.74
	≥1 yr	50.40 ± 15.62	50.40 ± 18.35	56.96 ± 16.33	58.72 ± 15.93	64.06 ± 14.85	50.40 ± 18.35
	p-value	0.38	0.002	0.18	0.07	0.04	0.85

Data presented as Mean± standard deviation.

FertiQoL: Fertility quality of life

** Student t-test

**Table IV:** Factor loadings from the FertiQoL principal component analysis

**Item of FertiQoL ** *	**Factor**					
	**1**	**2**	**3**	**4**	**5**	**6**
Q4	0.70	0.11	0.09	0.06	0.02	0.18
Q7	0.62	0.13	0.31	0.14	0.11	0.04
Q8	0.72	0.10	0.04	0.02	0.14	0.04
Q9	0.71	0.01	0.04	0.06	0.008	0.08
Q16	0.76	0.11	0.12	0.13	0.007	0.08
Q23	0.69	0.01	0.21	0.07	0.07	0.15
Q1	0.006	0.66	0.11	0.07	0.08	0.007
Q2	0.011	0.69	0.22	0.10	0.02	0.07
Q3	0.04	0.65	0.27	0.25	0.13	0.08
Q12	0.11	0.67	0.31	0.07	0.05	0.08
Q18	0.22	0.59	0.21	0.09	0.08	0.08
Q24	0.12	0.35	0.19	0.08	0.12	0.29
Q11	0.15	0.07	0.54	0.48	0.16	0.07
Q15	0.01	0.07	0.57	0.001	0.14	0.19
Q19	0.30	0.10	0.70	0.15	0.08	0.07
Q20	0.15	0.05	0.62	0.14	0.13	0.14
Q21	0.04	0.04	0.50	0.48	0.14	0.28
Q5	0.15	0.21	0.007	0.65	0.03	0.07
Q10	0.30	0.07	0.004	0.41	0.29	0.20
Q13	0.37	0.13	0.04	0.59	0.03	0.04
Q14	0.09	0.01	0.05	0.73	0.30	0.04
Q17	0.14	0.39	0.15	0.51	0.25	0.03
Q22	0.34	0.12	0.12	0.39	0.34	0.03
T2	0.20	0.05	0.07	0.29	0.29	0.34
T5	0.03	0.01	0.04	0.12	0.36	0.40
T7	0.07	0.05	0.14	0.35	0.31	0.42
T8	0.00	0.12	0.03	0.07	0.005	0.83
T9	0.008	0.2	0.04	0.08	0.00	0.82
T10	0.08	0.005	0.07	0.12	0.04	0.79
T1	0.29	0.07	0.05	0.20	0.34	0.29
T3	0.03	0.01	0.04	0.12	0.40	0.36
T4	0.31	0.35	0.14	0.05	0.42	0.07
T6	0.02	0.09	0.17	0.20	0.51	0.31

* Item numbers refer to question number in the original questionnaire.

## Discussion

The results show that the Iranian version of FertiQoL is a valid and reliable measure for assessing infertility problems and the effects of treatment on QoL of infertile patients .The results of discriminate validity indicate that the dimensions in FertiQoL can differentiate well for quality of life between patient groups based on the number of children and duration of infertility. According to the results of our study, patients with more children and shorter duration of infertility have better quality of life scores in all domains of FertiQoL. The results of the present study are similar to Karabulut and colleagues using Turkish version showed a longer duration of infertility was related to lower scores in the domain mind/body, social, tolerability and the total score of the questionnaire (p<0.05) (17). Moreover, the results of Boivin and co-worker showed that the score of core FertiQoL was significantly lower in patients without children compared with those with children (p<0.001) (7). 

In the present study, interval reliability of FertiQoL was reported desirable and all dimensions had α>0.7. However, in a study conducted in Taiwan, Cronbach’s level was unfavorable in the domains of relational, social and environmental which suggests that in Taiwan and China, perhaps due to the cultural differences with the English version of FertiQoL, it may be better to removed Q13 and T5 questions so that internal consistency is improved (18). The results of Boivin and co-worker indicated a desirable reliability with Cronbach’s’ alpha greater than 0.7 for all dimensions of FertiQoL (7). The reliability of Iranian version of FertiQoL was desirable (0.72-0.91).

The results of present study showed that Iranian version of FertiQoL is six-factorial with factor loading more than 0.3 for each factor. Item loading of the present study is similar to study of Boivin and co-worker (7). Although in Boivin and co-worker study, all questions had factor loading of more than 0.3 and preserved the questions Q1 (concentration) and Q2 (lie on hold) of mind/ body dimensions, Q10 (isolation) and Q13 (shame) of social dimension showed a cross-loading effect. In the study conducted in Taiwan, it is also reported that exclusion of question Q13 leads to improvement of Cronbach’s alpha of social dimension (10). In Maroufizadeh and co-worker study*,* all factor loadings were significant, except for Q15 and T2 (8). These findings indicated that some modifications for these items might be needed in the scale to yield better internal consistency. A cross-cultural difference might cause to these differences.

FertiQoL dimensions showed an intermediate correlation with similar dimensions of SF12 and HADS. Aarts and co-worker reported that there is a significant correlation between the Dutch version of FertiQoL and the scores of HADS questionnaire (0.29-0.71) indicating desirable convergent validity of FertiQoL (19). 

Our finding showed that there is a poor floor and ceiling effect in all dimensions of FertiQoL compared with SF12. The lower prevalence of floor and ceiling effect may be due to the fact that a tool is more responsive to clinical changes and it is suggested that general tools with their specific dimensions are less than specific tools. It is suggested that if the distribution of scores of a tool is more homogenous and less floor and ceiling effects exist, the tool is capable of better measurement (9, 20). More studies are recommended to evaluate the responsiveness FertiQoL. In Maroufizadeh and co-worker there are significant correlations between Core FertiQoL and Satisfaction with Life Scale. Moreover, the Core FertiQoL and its subscales negatively correlated with anxiety and depression (8). 

Generally, the results of the present study show that the Iranian version of FertiQoL has a six factorial construction with convergent validity, discrimination validity and a desirable reliability. Present study sample size is 300 participates considering 5 patients were necessary for each item (subject-to-item ratio: 5:1). However, the FertiQoL have 36 questions and at least 180 participate are needed for evaluation of psychometrics properties. Therefore our results have high statistical power and more reliable than previous studies. 

**Limitation**

There are some limitations to our study. The present study is limited to the patients recruited from the only referral hospital for infertility clinic; this may limit the generalizability of our findings. Furthermore, all of the participants in this study were married for cultural reasons in Iran. Although FertiQoL is a tool for measurement of quality of life in men and women, this study was conducted only on women. More studies on other psychometric properties of FertiQoL such as responsiveness, longitudinal validity and minimal important change are recommended.

## Conclusion

The Iranian version of FertiQoL is recommended to be used in Iran for assessment of quality of life of infertile patients in research, clinical environments and for evaluation of treatment success. The Iranian version of FertiQoL is a valid and reliable tool for measuring overall quality of life and several aspects of specific quality of life in infertile patients.
